# Profiling and Association of Microbiota and Volatile Compounds in Commercial Fermented Shrimp Pastes (*Terasi*)

**DOI:** 10.3390/foods15101623

**Published:** 2026-05-07

**Authors:** Reggie Surya, Ervina Ervina, Kantiya Petsong, David Nugroho

**Affiliations:** 1 Food Technology Department, Faculty of Engineering, Bina Nusantara University, Jakarta 11480, Indonesia; 2 Department of Food Technology, Faculty of Technology, Khon Kaen University, Khon Kaen 40002, Thailand; 3 Department of Integrated Science, Faculty of Science, Khon Kaen University, Khon Kaen 40002, Thailand

**Keywords:** fermentation, Indonesia, shrimp paste, *terasi*

## Abstract

*Terasi*, or fermented shrimp paste, is a staple condiment in Indonesian cuisine, produced through spontaneous fermentation of small crustaceans under high-salt conditions. Despite its widespread culinary use, comprehensive studies examining both the microbiota and volatile compounds in commercial *terasi* remain scarce. This study aimed to characterize the microbial composition and volatile profiles of ten commercial *terasi* products sourced from different regions of Indonesia, representing both traditional home industries and large-scale manufacturers. Culture-dependent microbial enumeration and 16S rRNA gene sequencing were employed to assess microbial diversity, while volatile compounds were identified and quantified using gas chromatography–mass spectrometry (GC-MS). Results revealed significant differences in microbial load and community composition among samples, with traditional products showing higher viable counts and microbial diversity, dominated by genera such as *Tetragenococcus*, *Bacillus*, *Weissella*, and *Halanaerobium*. Industrial samples, by contrast, contained no detectable microorganisms, likely due to sterilization practices for extended shelf life. A wide range of volatiles, including sulfur compounds, short-chain fatty acids, and trimethylamine, were identified across all samples, with a total of 48 detected compounds. Notably, correlation analysis revealed strong associations between specific bacterial genera and key volatile compounds, suggesting that microbial activity plays a central role in shaping *terasi*’s flavor. This integrative analysis provides new insights into the microbial–chemical interactions underlying fermented shrimp paste and offers potential applications for product standardization, starter culture development, and culinary innovation.

## 1. Introduction

Fermented shrimp paste, locally known as *terasi* in Indonesia, is a traditional condiment widely used across Southeast Asia. It is typically produced by salting and fermenting small crustaceans such as shrimp (*Acetes* spp.) or krill, followed by sun-drying and aging. The fermentation process, which may last from several days to months, occurs spontaneously and depends on indigenous microbiota and environmental conditions, resulting in a product with high variability in flavor, aroma, and microbial composition [1]. The rich umami taste and distinctive pungent odor of *terasi* are essential to its culinary appeal, commonly used as a base in Indonesian dishes such as *sambal terasi*, *sayur asem*, and *nasi goreng* [2,3]. These characteristics arise from complex biochemical transformations driven by microbial metabolism during fermentation [4,5].

*Terasi* is one of the most widely used traditional condiments in Indonesia and remains an important component of daily cooking, street food, and small-scale foodservice sectors. Beyond its culinary relevance, *terasi* also provides proteins, peptides, minerals, and fermentation-derived metabolites that may contribute to its nutritional and sensory value [6]. In addition, *terasi* production represents an important economic activity in many Indonesian coastal regions, where small household producers and artisanal industries convert locally available shrimp or krill into value-added fermented products [7]. Despite its broad consumption and commercial importance, scientific information regarding the combined microbial and volatile characteristics of commercial *terasi* products remains limited. Therefore, a more detailed investigation is warranted to support quality improvement, product consistency, and preservation of this culturally significant fermented food.

The commercial production of *terasi* in Indonesia is highly diverse and largely decentralized, with regional differences in raw materials, fermentation duration, salt concentration, and processing methods [8]. Most products are still made by small- to medium-scale home industries using traditional practices, typically without standardized fermentation controls [9]. This contributes to inconsistency in product quality, sensory attributes, and microbiological safety. Although several studies have focused on either microbial succession or volatile profiles in similar fermented seafood products, such as *kapi* in Thailand, *bagoong* in the Philippines, and *ngari* in India, comparative analyses that integrate both microbial and chemical profiling in Indonesian *terasi* are notably lacking.

Microorganisms, especially halotolerant and proteolytic bacteria like *Tetragenococcus*, *Bacillus*, and *Halanaerobium*, play a central role in the fermentation of shrimp paste [10,11]. These microbes are responsible for hydrolyzing proteins and lipids into amino acids and fatty acids, which serve as precursors for various aroma-active volatile organic compounds (VOCs). Common volatiles include aldehydes, ketones, alcohols, esters, organic acids, and sulfur-containing compounds, many of which contribute to the characteristic aroma of shrimp paste [12,13]. While the general roles of these microbial groups have been understood, the specific correlations between microbial genera and volatile compounds in commercial *terasi* remain unexplored.

To date, studies examining Indonesian *terasi* have largely focused on either microbial communities or chemical composition in isolation, with limited integration of these datasets across products from different production scales. The present study addresses this gap by providing a combined assessment of microbial abundance using culture-dependent and 16S rRNA gene-based approaches alongside volatile compound profiling by GC–MS. By bringing these datasets together, the study enables an exploratory evaluation of co-occurrence patterns between dominant microbial genera and aroma-active compounds in commercial *terasi*. While the correlation analysis does not establish functional or mechanistic relationships, it represents a systematic effort to contextualize microbial composition within the chemical aroma landscape of *terasi*. This integrative framework contributes to a more holistic understanding of *terasi* fermentation and provides a reference point for future studies aimed at fermentation control, product quality assessment, and the preservation of *terasi* as a culturally significant fermented food.

## 2. Materials and Methods

### 2.1. Preparation and Sampling of Terasi

A total of ten fermented shrimp paste (*terasi*) samples were used in this study, as presented in Table 1. Sample T1 was produced in the laboratory according to the fermentation method as previously described [5], using controlled conditions and standardized raw materials. The remaining nine samples (T2–T9) were commercially sourced from traditional markets and retail stores in West Java, Indonesia, to represent the diversity of *terasi* products available to consumers. The commercial *terasi* samples were categorized into three groups based on production scale and labeling status: (1) three traditional samples without brand or registration (non-labeled, artisanal), (2) three traditional samples with brand labels and local registration numbers (small-scale registered), and (3) three industrial-scale samples with brand labels and national registration numbers (large-scale production). All samples were collected in sealed packaging and transported under ambient conditions to the laboratory. Upon arrival, each sample was homogenized and portioned for further chemical and microbial analysis. Ten *terasi* products were selected to represent a broad range of commercially available products differing in production scale, labeling status, and geographical origin. As an exploratory market survey study involving heterogeneous commercial samples, the sample size was designed to maximize product diversity rather than to estimate population prevalence.

### 2.2. Volatile Compound Analysis

Volatile compounds in the *terasi* samples were analyzed using an untargeted headspace solid-phase microextraction gas chromatography–mass spectrometry (HS-SPME-GC–MS) approach (GC-MS, model HP 5973, Agilent Technologies, Santa Clara, CA, USA) to comprehensively profile aroma-active compounds present in the samples as previously described [14] with some modifications. No fixed target panel of volatile compounds was pre-selected. Prior to analysis, approximately 2 g of homogenized *terasi* sample was placed in a 20 mL headspace vial and equilibrated at 60 °C for 30 min. Headspace solid-phase microextraction (HS-SPME) was performed using a DVB/CAR/PDMS fiber (Supelco, Bellefonte, PA, USA), which was exposed to the sample headspace for 30 min under the same temperature conditions. The fiber was then desorbed in the GC injection port at 250 °C in splitless mode. Separation was carried out using a DB-Wax capillary column (30 m × 0.25 mm i.d., 0.25 μm film thickness), with helium as the carrier gas at a constant flow rate of 1.0 mL/min. The oven temperature was programmed to start at 40 °C (held for 3 min), ramped to 200 °C at 5 °C/min, and held for 5 min. The mass spectrometer was operated in electron impact (EI) mode at 70 eV, scanning from *m*/*z* 35 to 500. Volatile compounds were identified by comparing the mass spectra with those in the NIST/EPA/NIH mass spectral library and confirmed by their retention indices, calculated using a series of n-alkanes analyzed under the same conditions. The identified volatile compounds were expressed as relative abundance.

### 2.3. Microbiological Analysis

For microbial enumeration [5], *terasi* samples (1 g each) were subjected to serial tenfold dilutions using sterile peptone water. Appropriate dilutions were plated onto selective and non-selective media to determine total viable counts, halophilic bacteria, and lactic acid bacteria populations. Specifically, standard Plate Count Agar (Thermo Fisher Scientific, Waltham, MA, USA) was used for total viable counts, halophilic agar supplemented with 25% NaCl (HiMedia Laboratories, Mumbai, India) was used for halophilic bacterial enumeration, and De Man, Rogosa and Sharpe (MRS) agar (HiMedia Laboratories, India) was used to quantify lactic acid bacteria. All inoculated plates were incubated at 35 °C for 120 h (5 × 24 h) under aerobic conditions prior to colony counting.

For microbial community profiling [15], total DNA was extracted from *terasi* samples using a commercial bacterial DNA extraction kit (Bacterial Miniprep Kit, Zymo Research, Irvine, CA, USA) following the manufacturer’s standard protocol. Amplification of the bacterial 16S rRNA gene was performed using universal primers targeting the V3–V4 regions. Amplicon sequencing was conducted on the Illumina MiSeq platform according to the manufacturer’s instructions to determine microbial abundance and diversity. The quality of extracted DNA was assessed using a NanoDrop spectrophotometer (Thermo Fisher Scientific, USA) by measuring absorbance ratios at 260/280 nm and 260/230 nm. DNA integrity was further confirmed by electrophoresis on 1% (*w*/*v*) agarose gels stained with SYBR Safe DNA gel stain (Invitrogen, Carlsbad, CA, USA), run at 100 V for 30 min. Only DNA samples with 260/280 ratios between 1.8 and 2.0 and clear high-molecular-weight bands were used for sequencing. Each biological sample was extracted in triplicate to ensure reproducibility and minimize technical variation. Sequencing data were processed using the QIIME2 pipeline (version 2022.2). Taxonomic assignment of bacterial sequences was performed using the SILVA rRNA database (version 138) at a 99% sequence similarity threshold.

### 2.4. Statistical Analysis

Data of microbial enumeration (n = 5) were analyzed evaluated using one-way Analysis of Variance (ANOVA), followed by Tukey’s Honestly Significant Difference (HSD) post hoc test to determine significant differences between fermentation stages (*p* < 0.05). The findings were expressed as mean ± standard deviation (SD). Heatmaps were generated using Displayr online platform. Pearson’s correlation coefficient (r) was calculated to determine the strength and direction of linear associations between the abundance of microbial genera and volatile compounds. All statistical analyses were performed using Systat 10 for Windows. In addition, principal component analysis (PCA) was performed to explore multivariate relationships among *terasi* samples based on dominant bacterial genera and volatile compounds. Prior to PCA, variables were standardized using z-score transformation to account for differences in scale. The analysis was used as an exploratory unsupervised approach to visualize clustering patterns and identify variables contributing most strongly to sample differentiation.

## 3. Results and Discussion

### 3.1. Volatile Compounds in Commercial Terasi Samples

**Figure 1:**
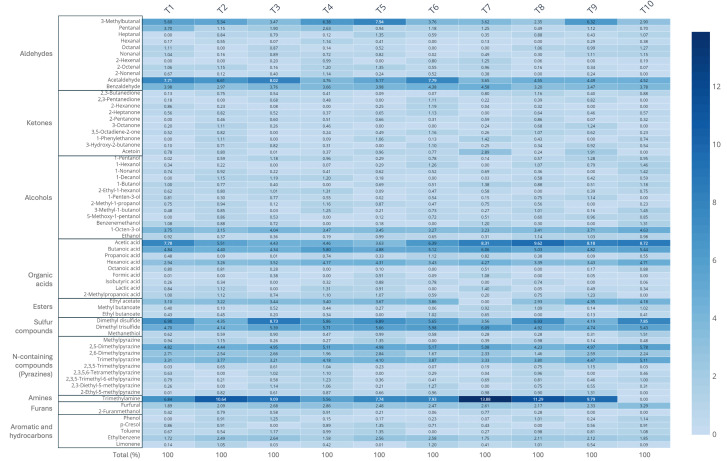
Relative abundance of volatile compounds detected in *terasi* samples (n = 3).

The analysis of volatile compounds in the ten commercial *terasi* samples revealed a diverse and complex chemical profile, with a total of 48 compounds identified across all products (Figure 1). A representative chromatogram is presented in Supplementary Figure S1. These volatiles, which include sulfur compounds, fatty acids, aldehydes, alcohols, esters, and pyrazines, are typically formed during fermentation through microbial metabolism, enzymatic degradation of proteins and lipids, and non-enzymatic reactions such as Maillard-type chemistry. Although the general composition was similar across samples, the relative abundance of individual compounds varied, likely reflecting differences in fermentation practices, raw materials, and microbial dynamics. This pattern is in line with previous studies on shrimp paste, where raw materials and production methods strongly influence aroma profiles [16,17,18].

A noteworthy finding was the dominance of trimethylamine (TMA) in several samples, where it contributed more than 10% of the total volatile composition. TMA is a well-known product of microbial reduction of trimethylamine N-oxide (TMAO), which is naturally present in marine organisms [19]. It imparts a strong fishy aroma that is characteristic of crustacean-based fermented products. The high levels of TMA observed in samples such as T2, T4, and T7 suggest intense microbial activity, possibly involving halophilic anaerobes like *Halanaerobium* or *Alkalibacillus*, which are known to thrive in high-salt environments and have been linked to TMA formation [20].

Sulfur-containing compounds, particularly dimethyl disulfide (DMDS) and dimethyl trisulfide (DMTS), were also present in significant amounts. These volatiles are typically formed during the degradation of sulfur-rich amino acids such as methionine and cysteine and are commonly associated with savory, cabbage-like, and slightly burnt notes [21]. Their presence was most prominent in samples like T3 and T5. These findings are consistent with earlier reports showing that sulfur volatiles play a central role in the flavor profile of fermented fish and shrimp products [22].

In addition, short-chain fatty acids, such as butanoic acid and hexanoic acid, were detected in most samples, often in appreciable amounts. These compounds are byproducts of lipid breakdown and amino acid deamination, processes typically carried out by proteolytic and lipolytic bacteria such as *Bacillus* spp. [23]. Their contribution to the aroma is significant, often described as cheesy, sweaty, or rancid—attributes that, when balanced, are desirable in traditionally fermented seafood [24]. Their presence was mostly detected in samples T2, T5, and T6.

Interestingly, several samples also contained pyrazines, including 2,5-dimethylpyrazine and trimethylpyrazine, which are typically associated with roasted or nutty notes [25]. Although these compounds are commonly linked to heat-induced reactions like the Maillard reaction [26], they can also be synthesized by certain bacteria such as *Bacillus subtilis* during fermentation [27]. Their detection in samples like T3 and T7 suggests that both thermal effects from sun-drying and microbial contributions may play a role in their formation. These compounds, while present in lower quantities than sulfur compounds or fatty acids, add additional complexity to the aromatic profile of *terasi*.

Although variations in the proportions of certain compounds were observed, the overall abundance and diversity of volatiles were relatively similar between *terasi* from home industries (T1–T7) and those from large-scale producers (T8–T10). This finding implies that key volatile components are primarily generated during the early stages of fermentation and tend to persist even after heat treatments or sterilization, which are commonly applied in industrial processing to extend shelf life. It also highlights the importance of the fermentation stage in defining the sensory characteristics of *terasi*, regardless of production scale. Understanding how microbial activity and processing conditions influence volatile profiles can inform quality control strategies while preserving the traditional flavor of this culturally significant condiment.

### 3.2. Microbial Load and Diversity in Commercial Terasi Samples

**Figure 2:**
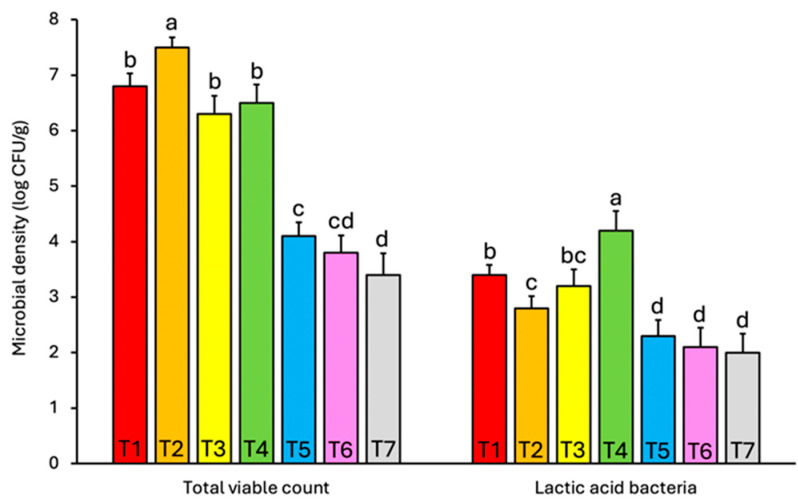
Microbial load in *terasi* samples (T1–T7). Data (n = 3) are expressed as mean ± SD. Different letters in a group indicate significant difference (*p* < 0.05) following one-way Anova and Tukey’s HSD post hoc test. No microbial load was detected in samples T8–T10.

Microbial enumeration revealed that only samples T1–T7, all derived from small- to medium-scale home industries, showed detectable microbial populations, whereas T8–T10, representing large-scale industrial products, exhibited no cultivable microbial load (Figure 2). This is consistent with previous findings indicating that high salt concentration, extended drying, and thermal treatments used in industrial fermentation processes significantly reduce or eliminate viable microorganisms in fermented products [28].

Indeed, commercial *terasi* produced by large-scale industries commonly undergo post-fermentation stabilization processes designed to improve shelf life, microbiological safety, and product consistency. These processes may include thermal heating, roasting, steam treatment, or pasteurization, which can markedly reduce viable microbial populations. In addition, industrial products are often subjected to more intensive drying, resulting in lower water activity that further limits microbial survival and growth during storage. Vacuum or sealed packaging may also reduce oxygen availability and minimize post-processing contamination. Similar stabilization strategies have been reported in fermented seafood industries, where heating and moisture reduction are applied to extend shelf life and maintain product quality [29]. Therefore, the absence of detectable microbial counts in samples T8–T10 is likely attributable to a combination of thermal inactivation, low water activity, and protective packaging rather than the complete absence of microorganisms during earlier fermentation stages.

The microbial enumeration results revealed that total viable count (TVC) and lactic acid bacteria (LAB) counts were notably lower in samples T5–T7 compared to T1–T4 (Figure 2). This variation is likely attributed to differences in processing practices, particularly in terms of fermentation duration, salt concentration, and post-fermentation handling. Samples T5–T7 were all commercially packaged products with registered product identification numbers from local health authorities (Dinas Kesehatan), suggesting some level of standardization or hygienic control that may include partial pasteurization, higher salt use, or drying at elevated temperatures. These treatments can significantly reduce microbial populations. In contrast, T1–T4 were unbranded products sourced from traditional markets, likely produced using more artisanal methods with minimal intervention, allowing for more active and prolonged microbial fermentation.

**Figure 3:**
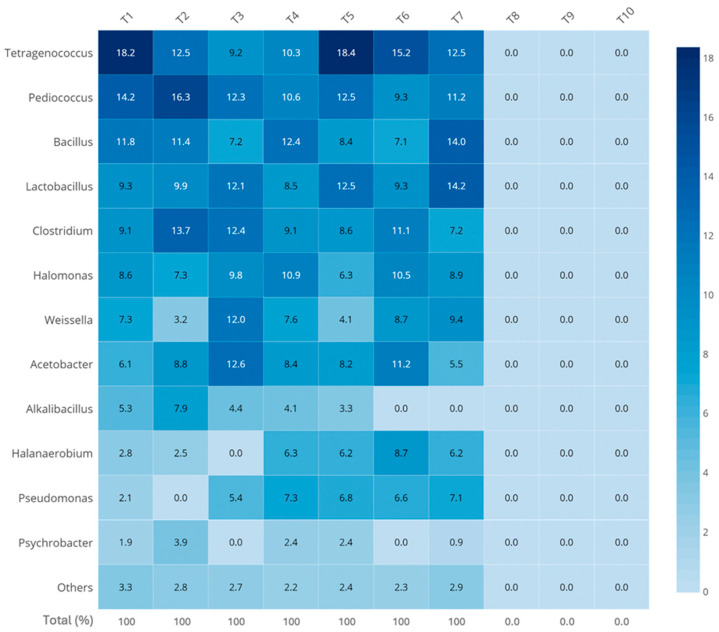
Relative abundance of bacterial genera in *terasi* samples (n = 3).

The 16S rRNA gene sequencing results revealed a rich and diverse bacterial community, though composition varied significantly across samples (Figure 3). At the phylum level, Firmicutes and Proteobacteria were dominant in most products, aligning with previous reports from other fermented seafoods [30,31]. In samples T1–T4, *Tetragenococcus*, *Weissella*, and *Bacillus* were among the most abundant genera. These bacteria are commonly found in fermented fish products and play critical roles in proteolysis, acidification, and the generation of flavor-active metabolites such as organic acids and sulfur compounds [32]. Their prevalence in these samples supports the high volatile compound complexity.

The predominance of *Tetragenococcus* in several traditional samples is biologically relevant, as this halophilic lactic acid bacterium is widely recognized as a key functional microorganism in high-salt fermented seafoods such as fish sauce, shrimp paste, and soy-based fermentations. *Tetragenococcus* spp. are capable of carbohydrate fermentation, acid production, osmotic stress adaptation, and amino acid metabolism, thereby contributing to pH stabilization and flavor precursor generation. Previous studies have frequently reported *Tetragenococcus halophilus* as a desirable microorganism associated with quality fermentation in salted seafood products [33].

Likewise, the abundance of *Bacillus* spp. may be associated with their strong extracellular protease and lipase activities. These enzymes hydrolyze proteins and lipids into peptides, amino acids, and free fatty acids, which subsequently act as precursors for aldehydes, ketones, esters, pyrazines, and short-chain fatty acids. *Bacillus* species are commonly reported as technologically important microorganisms in many protein-rich fermented foods [34].

In contrast, *Halanaerobium* and *Alkalibacillus* were more prominent in samples T5–T7, suggesting a shift toward halophilic and anaerobic microbial communities. These genera have been frequently reported in high-salt fermentations and are capable of producing distinct volatiles such as trimethylamine and sulfides [35]. Their abundance may reflect more controlled or prolonged fermentation conditions, where oxygen depletion and salt accumulation allow these microbes to outcompete others. Notably, their presence correlated well with the dominant aroma compounds in these samples, especially sulfur- and amine-based volatiles [35].

As expected, samples T8–T10 yielded no detectable bacteria in both culture-based and sequencing analyses, likely due to the applied sterilization process. These industrial products, while microbiologically stable, lacked many of the complex bacterial taxa found in traditionally made *terasi*. This observation supports the notion that microbial diversity—and by extension, microbial metabolism—is closely tied to the authenticity and richness of flavor in fermented fish products [15,32]. While industrial processing ensures safety and shelf stability, it may limit the dynamic biochemical transformations typically driven by living microbial communities.

Alpha diversity analysis further quantified these differences in microbial community structure (Supplementary Figure S2). Traditional *terasi* samples (T1–T7) exhibited higher observed amplicon sequence variants (ASVs) and Chao1 indices, indicating greater microbial richness compared to industrial products. The Shannon diversity index was also higher in traditional samples, reflecting a more even distribution of microbial taxa. In contrast, industrial *terasi* (T8–T10) showed near-zero values across all diversity indices, consistent with the absence of cultivable microorganisms and detectable bacterial taxa following sterilization. These results corroborate both culture-based enumeration and taxonomic profiling, demonstrating that industrial processing markedly reduces microbial diversity.

Overall, the results emphasize that microbial load and diversity in *terasi* are highly dependent on production scale and post-fermentation treatment. The home industry products, especially T1–T4, preserved a more diverse and active microbiota, likely contributing to more intense and varied flavor profiles. In contrast, industrial samples offered microbial safety and consistency but at the cost of microbial-driven complexity. Understanding this balance is crucial for both maintaining traditional practices and improving quality control in the modernization of fermented seafood products.

### 3.3. Correlation Between Microbiota and Volatile Compounds

**Figure 4:**
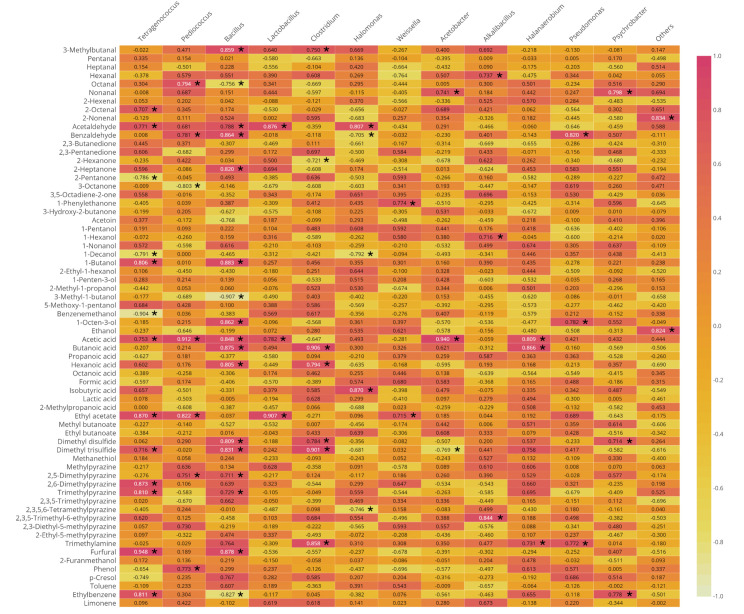
Correlation strength analysis of bacterial genera and volatile compounds. Each cell represents a Pearson’s correlation coefficient between a genus and a volatile compound (value −1.0 to 1.0). Asterisk signs (*) represent significant correlations (*p* < 0.05).

Correlation analysis was performed to explore potential associations between microbial community composition and volatile compound profiles across commercial *terasi* samples. While previous studies have commonly examined microbial communities or volatile metabolites independently, this study integrates both datasets to provide an exploratory assessment of genus–volatile relationships. A Pearson’s correlation matrix (Figure 4) was constructed using dominant bacterial genera and key aroma-active compounds, allowing patterns of co-occurrence to be evaluated across samples. This approach offers a data-driven framework for examining potential links between microbiota composition and aroma-related metabolites without implying causality.

Several notable positive associations were observed. The genus *Tetragenococcus* showed positive associations with dimethyl disulfide, dimethyl trisulfide, and 3-methylbutanal, compounds commonly linked to savory and pungent aromas in fermented foods and typically derived from sulfur-containing and branched-chain amino acid metabolism [36,37]. Similar associations between *Tetragenococcus* and sulfur- and malty-related volatiles have been reported in fermented seafood matrices, as neo-formed products of microbial catabolism of sulfur-containing amino acids such as methionine and cysteine [38]. While the present correlation analysis does not demonstrate functional activity, the observed associations are consistent with the established metabolic potential of *Tetragenococcus* described in previous studies. Notably, *Tetragenococcus* has been widely explored as a starter culture in controlled fermentations of salted seafood products [39], highlighting its relevance as a target organism for future mechanistic and application-oriented studies rather than a conclusion drawn from the present dataset.

*Bacillus* spp. exhibited positive correlations with butanoic acid, hexanoic acid, and ethyl acetate, volatiles that contribute cheesy, rancid, and fruity notes and are frequently detected in high-protein fermentations [34]. These associations were particularly evident in samples produced by traditional home industries, where *Bacillus* abundance and volatile intensity tended to be higher. The observed patterns are consistent with known metabolic capabilities of *Bacillus* reported in other fermented foods, although functional contributions cannot be confirmed based on correlation analysis alone.

The halophilic and anaerobic genus *Halanaerobium* showed a positive association with trimethylamine, a compound commonly regarded as a marker of marine protein degradation and a contributor to strong fishy odors [40]. *Halanaerobium* has previously been reported in high-salt, anaerobic fermentation systems and linked to the production of amine- and sulfur-containing compounds [41]. Its correlation with trimethylamine in this study suggests a potential association with aroma development under high-salt conditions, warranting further investigation. Under anaerobic saline environments, members of this genus may utilize trimethylamine N-oxide (TMAO) as an electron acceptor, resulting in trimethylamine formation and characteristic marine fishy odors. Similar patterns have been documented in fermented fish products and fish sauces [42].

*Weissella* showed positive associations with ethyl acetate and 1-octen-3-ol, volatile compounds contributing fruity and mushroom-like notes to fermented foods. Although *Weissella* is less commonly reported in seafood fermentations than other lactic acid bacteria, its presence has been documented in various fermented food matrices and is known to be associated with diverse metabolic activities, including ester formation and vitamin production [43]. In the present study, the observed correlations suggest a potential association between *Weissella* abundance and specific volatile compounds; however, these relationships should be interpreted cautiously, as correlation analysis does not confirm functional involvement.

**Figure 5:**
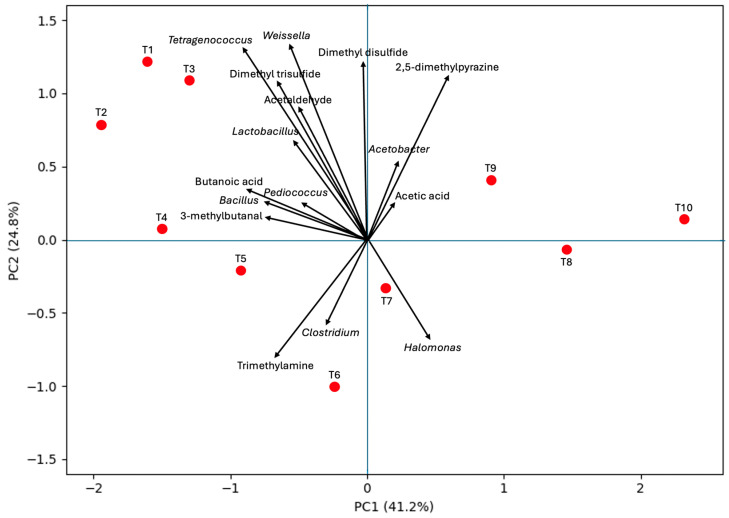
PCA biplot of *terasi* samples based on dominant bacterial genera and volatile compounds. Samples positioned close together indicate greater similarity in microbial–volatile profiles, whereas variables with longer vectors contributed more strongly to separation among samples.

To complement the correlation analysis, principal component analysis (PCA) was performed as an unsupervised multivariate approach to visualize overall relationships among *terasi* samples, dominant bacterial genera, and volatile compounds (Figure 5). The first two principal components explained 66% of the total variance (PC1 = 41.2%, PC2 = 24.8%), indicating that these axes captured most of the variation among products. A clear separation trend was observed along PC1, where traditional products (T1–T7) were generally distributed on the negative side, whereas industrial products (T8–T10) were positioned on the positive side. This pattern suggests that production scale and associated processing practices substantially influenced the combined microbial–metabolite profiles of the products. Traditional samples were more closely associated with fermentative and halophilic bacterial genera such as *Tetragenococcus*, *Weissella*, *Lactobacillus*, *Pediococcus*, and *Bacillus*, together with sulfur-containing compounds (dimethyl disulfide, dimethyl trisulfide), aldehydes, and butanoic acid. These metabolites are commonly linked to amino acid degradation, proteolysis, and active microbial fermentation, indicating a more microbiologically dynamic fermentation process in artisanal products.

In contrast, industrial samples T8–T10 were clearly separated from most traditional products and showed weaker associations with fermentation-related bacteria. Samples T9 and T10 were oriented toward acetic acid and 2,5-dimethylpyrazine, suggesting a greater influence of processing-derived reactions, oxidative changes, or heat-associated aroma formation rather than ongoing microbial metabolism. Sample T8 appeared relatively isolated near the PC1 axis, indicating a comparatively neutral or simplified profile. Meanwhile, samples T6 and T7 were positioned closer to trimethylamine, *Clostridium*, and *Halomonas*, implying that certain commercial products retained characteristics associated with halophilic or amine-producing microbial activity. Overall, the PCA supports the view that traditional *terasi* products retain more diverse fermentation-driven microbial and metabolite signatures, whereas industrial products exhibit more standardized profiles likely shaped by post-fermentation treatments such as drying, heating, or sterilization.

Comparable microbial and volatile patterns have been reported in related fermented seafood products from other regions. Thai *kapi*, Chinese shrimp paste, and traditional fish sauces are frequently characterized by the dominance of *Tetragenococcus*, *Bacillus*, and other halophilic taxa, together with abundant amines, sulfur compounds, fatty acids, and Maillard-derived volatiles [44,15,^45^]. Despite regional differences in raw materials and processing practices, these products share common ecological drivers, including high salt concentrations, protein-rich substrates, and reduced oxygen availability, which select for microorganisms with similar functional traits. The present findings therefore support the broader concept that microbial ecology is a major determinant of aroma development across fermented seafood products [46].

The variability observed among commercial *terasi* samples may also be influenced by several production-related factors beyond microbial composition alone. Differences in salt concentration, fermentation duration, ambient temperature and humidity, raw material quality, and manufacturer-specific processing practices (e.g., mixing frequency, container type, drying conditions, and hygiene management) are known to affect both microbial succession and metabolite formation during fermented seafood production [47]. Because the present study examined commercially available products from multiple producers, such metadata were not consistently available and therefore could not be incorporated into the present analysis. Consequently, the differences detected among samples should be interpreted as the result of combined manufacturing and ecological factors rather than single-variable effects.

Overall, the integrative analysis conducted in this study highlights the value of combining microbial community profiling with volatile compound characterization to move beyond descriptive cataloguing of commercial *terasi* products. While reduced microbial presence in industrially processed products may be expected, this work provides the first systematic comparison linking production scale, microbial community structure, and aroma-associated metabolites within a single analytical framework. Importantly, the correlation analysis enables the identification of specific microbial genera associated with key volatile compounds, offering insight into potential functional roles of dominant taxa in aroma formation without assuming causality. To the best of our knowledge, such genus–volatile association patterns have not previously been reported for *terasi*, and they provide a data-driven foundation for hypothesis development, targeted fermentation control, and future mechanistic studies aimed at improving product quality while preserving microbial and cultural authenticity. Furthermore, this study could support fermented seafood products and their by-products as functional food ingredients and delivery systems [48,49].

### 3.4. Limitations of the Study

Several limitations of this study should be acknowledged. First, the number of commercial *terasi* samples examined was limited, which reduced statistical power, particularly for correlation analyses between microbial taxa and volatile compounds. Therefore, the observed associations should be interpreted as exploratory and hypothesis-generating rather than as evidence of causal relationships. Second, although analytical measurements included technical replication, the number of biological replicates within each production category was constrained by the limited availability and inherent heterogeneity of commercial *terasi* products, which may restrict broader generalization. Third, the study relied on 16S rRNA gene sequencing and volatile compound profiling without functional validation of microbial contributions to specific aroma compounds. In addition, DNA-based sequencing detects total microbial signatures and cannot distinguish between live, inactive, and dead cells, as residual DNA may persist after microbial inactivation. This limitation is particularly relevant for industrial samples that may have undergone heat treatment or sterilization. Also, product labels did not consistently disclose salt concentration, fermentation duration, drying conditions, or raw material ratios. These uncontrolled variables may have contributed to sample-to-sample heterogeneity and limited causal interpretation. Finally, because this study used a cross-sectional design based on finished commercial products, it provides only a snapshot of product characteristics rather than controlled fermentation dynamics, which limits mechanistic interpretation. Future studies using larger sample sets, controlled fermentation models, viability-targeted microbial analyses, functional assays, and sensory evaluation would help strengthen statistical robustness and deepen mechanistic understanding.

## 4. Conclusions

The present study provides a comparative characterization of microbial communities and volatile compound profiles in commercial *terasi* products produced at different scales. Traditional products exhibited higher microbial richness and greater diversity of aroma-active metabolites, whereas industrial products, while consistent and shelf-stable, showed minimal microbial presence and reduced chemical complexity. Dominant bacterial genera, including *Tetragenococcus*, *Bacillus*, and *Halanaerobium*, were consistently associated with specific volatile compounds such as trimethylamine, dimethyl disulfide, and short-chain fatty acids. These associations, derived from correlation analysis, should be interpreted as exploratory and reflect co-occurrence patterns rather than direct evidence of microbial functionality.

By integrating culture-based enumeration, 16S rRNA gene sequencing, and volatile profiling, this study extends existing descriptive knowledge by providing a data-driven framework for examining potential relationships between microbiota composition and aroma-related metabolites in *terasi*. Collectively, these results suggest that dominant halophilic microbiota may function not only as taxonomic markers of fermentation, but also as important contributors to aroma generation and product differentiation in commercial *terasi*. Although the findings do not establish mechanistic links, they generate testable hypotheses regarding the involvement of specific microbial taxa in aroma development during fermented shrimp paste production.

From an industrial perspective, the present findings may support the development of controlled fermentation strategies, including the use of selected starter cultures such as *Tetragenococcus* or beneficial *Bacillus* strains, to improve product consistency while preserving desirable traditional flavor characteristics. In addition, characterization of volatile profiles may assist manufacturers in designing products that better align with consumer preferences for specific aroma notes. Therefore, future optimization of *terasi* production should aim to balance safety, consistency, and sensory authenticity.

Future studies should build upon these observations by incorporating controlled fermentation experiments, functional assays, and sensory evaluation to validate the contributions of individual microbial taxa to flavor formation and consumer perception. In addition, systematic evaluation of fermentation parameters and safety-related markers, including biogenic amines, would support efforts to improve product consistency and safety while maintaining the traditional characteristics of *terasi*. The application of selected starter cultures, such as beneficial halophilic or proteolytic strains, may further enable more consistent fermentation performance and targeted aroma development. Likewise, integration of volatile profile data with consumer preference studies could assist manufacturers in designing products tailored to specific market segments, including premium, regional specialty, or export-oriented *terasi* products. Together, these approaches may facilitate the modernization of *terasi* production in a manner that respects both cultural heritage and quality standards, while enhancing industrial competitiveness and consumer appeal.

## Figures and Tables

**Table 1:** List of *terasi* samples used in the study.

Sample Code	Sampling Location	Location of Manufacturer	Note
T1	Laboratory	Tangerang, Banten	Made for the study by the authors
T2	Traditional market	Cirebon, West Java	Home industry product, sold commercially with no brand
T3	Traditional market	Banyumas, Central Java	Home industry product, sold commercially with no brand
T4	Traditional market	Brebes, Central Java	Home industry product, sold commercially with no brand
T5	Traditional market	Indramayu, West Java	Home industry product, sold commercially with brand and registered product identification number issued by a local authority (Dinas Kesehatan)
T6	Traditional market	Rembang, Central Java	Home industry product, sold commercially with brand and registered product identification number issued by a local authority (Dinas Kesehatan)
T7	Minimarket	Pangkalpinang, Bangka	Home industry product, sold commercially with brand and registered product identification number issued by a local authority (Dinas Kesehatan)
T8	Minimarket	Mojokerto, East Java	Large-scale industry product, sold commercially with brand and registered product identification number issued by the national authority (BPOM)
T9	Supermarket	Bekasi, West Java	Large-scale industry product, sold commercially with brand and registered product identification number issued by the national authority (BPOM)
T10	Supermarket	Karawang, West Java	Large-scale industry product, sold commercially with brand and registered product identification number issued by the national authority (BPOM)

## Data Availability

The original contributions presented in this study are included in the article/Supplementary Materials. Further inquiries can be directed to the corresponding author.

## Supplementary Materials

The following supporting information can be downloaded at: https://www.mdpi.com/article/10.3390/foods15101623/s1, Figure S1: Representative HS-SPME–GC–MS total ion chromatogram of volatile compounds detected in traditional terasi sample (T1); Figure S2: Box plots showing alpha diversity indices of microbial communities in terasi samples: observed amplicon sequence variants (ASVs), Chao1 richness estimator, and Shannon diversity index for traditional and industrial terasi samples. Boxes represent the interquartile range with the median, and whiskers indicate the minimum and maximum values.
