# Supplementary material for: Profiling and Association of Microbiota and Volatile Compounds in Commercial Fermented Shrimp Pastes (Terasi)
Source: Foods. 2026 May 7;15(10):1623. doi: 10.3390/foods15101623 (PMC13205825; doi:10.3390/foods15101623)
Supplement: Supplementary file 1 [file foods-15-01623-s001.zip › foods-4274486-supplementary.pdf]

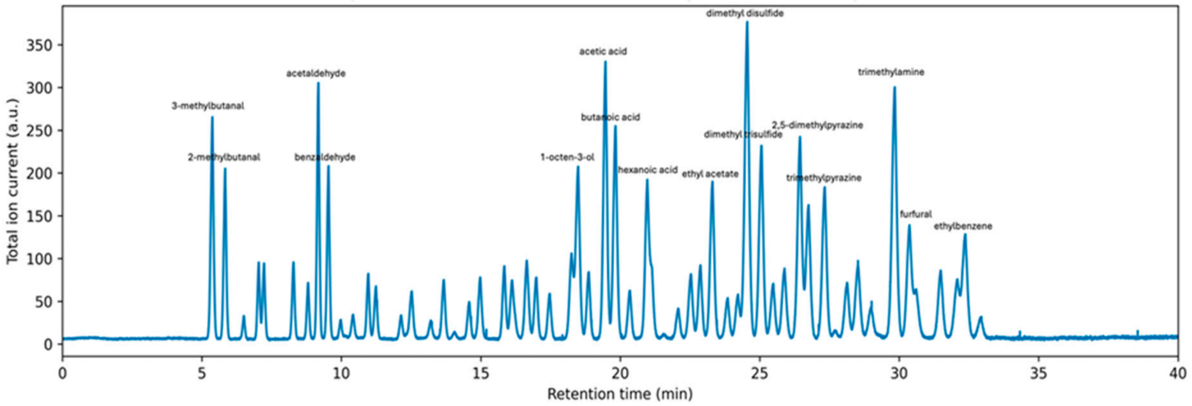

Supplementary Figure S1. Representative HS-SPME–GC–MS total ion chromatogram of volatile compounds detected in traditional *terasi* sample (T1).

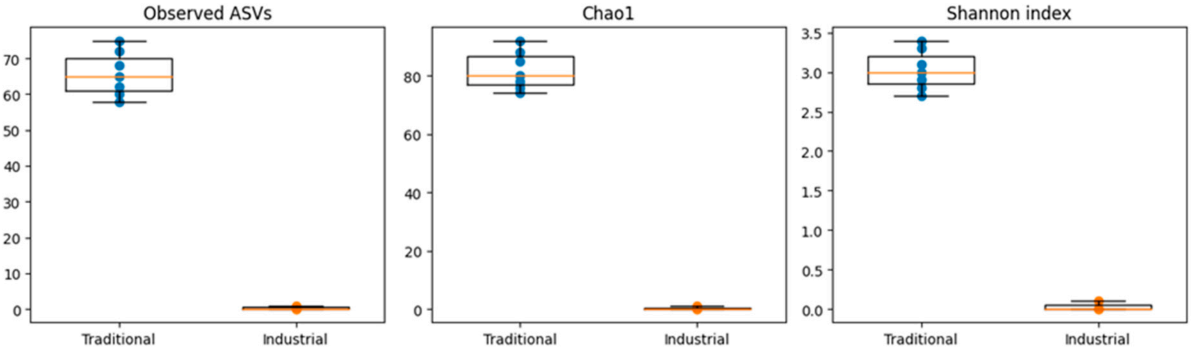

Supplementary Figure S2. Box plots showing alpha diversity indices of microbial communities in *terasi* samples: observed amplicon sequence variants (ASVs), Chao1 richness estimator, and Shannon diversity index for traditional and industrial *terasi* samples. Boxes represent the interquartile range with the median, and whiskers indicate the minimum and maximum values.
